# Evolutionary approach to violating group anonymity using third-party data

**DOI:** 10.1186/s40064-016-1692-9

**Published:** 2016-01-26

**Authors:** Dan Tavrov, Oleg Chertov

**Affiliations:** National Technical University of Ukraine “Kyiv Polytechnic Institute”, Kyiv, Ukraine

**Keywords:** Group anonymity, Privacy-preserving data publishing, Fuzzy inference, Memetic algorithm, Microfile, Subgroup discovery

## Abstract

In the era of Big Data, it is almost impossible to completely restrict access to primary non-aggregated statistical data. However, risk of violating privacy of individual respondents and groups of respondents by analyzing primary data has not been reduced. There is a need in developing subtler methods of data protection to come to grips with these challenges. In some cases, individual and group privacy can be easily violated, because the primary data contain attributes that uniquely identify individuals and groups thereof. Removing such attributes from the dataset is a crude solution and does not guarantee complete privacy. In the field of providing individual data anonymity, this problem has been widely recognized, and various methods have been proposed to solve it. In the current work, we demonstrate that it is possible to violate group anonymity as well, even if those attributes that uniquely identify the group are removed. As it turns out, it is possible to use third-party data to build a fuzzy model of a group. Typically, such a model comes in a form of a set of fuzzy rules, which can be used to determine membership grades of respondents in the group with a level of certainty sufficient to violate group anonymity. In the work, we introduce an evolutionary computing based method to build such a model. We also discuss a memetic approach to protecting the data from group anonymity violation in this case.

## Background

A son of Dmitrii Mendeleyev, the world-renowned chemist and creator of the periodic table, recalls (Tishchenko and Mladientsev 1993, pp. 353–354) an interesting fact. In 1890, his father came up with a formula of the smokeless pirokollody gunpowder (Gordin 2003), which at the time was thoroughly protected by French manufacturers. As it turned out, Mendeleyev’s findings were based on analyzing public statistical data from the railroad company annual report on freight traffic. A separate branch line supplied the gunpowder factory. Annual statistics provided all the necessary information to easily retrieve the gunpowder composition ratios.

One hundred and twenty five years later, in the era of Big Data, various statistical data are publicly available. The task of ensuring that security intensive information does not leak out becomes much more challenging. A modern man lives and works in a society oriented toward collecting and storing data on each and every person. Statistics services do that (census forms), taxing services do that (tax declarations), medical facilities do that (patient’s medical records), law enforcement agencies do that (person’s IDs), employers do that (CVs), retail stores do that (personal discount cards), security does that (security cameras files), and so on and so forth. Problems of preserving privacy in such data are widely discussed within the field of privacy-preserving data publishing (Fung et al. 2010; Wong and Fu 2010). To great extent, appropriate protection implies removing identifiers (passport data, full name etc.), and distorting the data (e.g., values of certain characteristics are swapped between respondents or get noised) or suppressing them (e.g., data on elder people are grouped in a category of *senior citizens*).

At the same time, problems of protecting *group* distributions for certain categories of respondents remain unsolved. Let us consider a case when abnormal concentration of nuclear physicists on a specific territory reveals the site of a secret nuclear research facility. Of course, removing such attributes as *Occupation* or *Industry* seems to be a first choice. However, the risk of privacy violation remains high if there is information about where respondents pursued their higher education (e.g., National Institute for Nuclear Science and Technology for academic training in atomic energetics is situated in Saclay commune, France), or about where they lived (for instance, Dubna, Russian Federation, is a home to Joint Institute for Nuclear Research). Therefore, the task of protecting distributions for a certain group of respondents (which can be persons, households, enterprises etc.) with minimal distortion of primary statistical data is a pressing one.

There are numerous practical cases when we do not have attributes at our disposal that classify a respondent as belonging to a certain group (either because they were deliberately removed by the data publisher, or because they were not present in the first place). However, we can try to restore group distributions by analyzing publicly available data such as statistical surveys, polls etc. (Chertov and Tavrov 2015). Using expert judgments about these data, we can build a fuzzy model of a group in a form of a fuzzy inference system (FIS) that, for each respondent, gives her membership grade in the group under consideration. A distribution constructed this way can violate group anonymity as discussed above.

Expert judgments often are not a reliable source of fuzzy rules that constitute the main part of any FIS. Sometimes, it is hard even to properly identify attributes necessary to include into a model of a group, let alone determine particular fuzzy rules. In this work, we propose an evolutionary based method of building the fuzzy model using third-party data. We also describe a memetic algorithm for solving the task of anonymizing the obtained distribution. This algorithm seeks minimal distortion in the microfile, and at the same time ensures that group anonymity cannot be violated.

## Related work

### Data anonymity

*Anonymity* of a subject means (Pfitzmann and Hansen 2010) that it is not identifiable (uniquely characterized) within a set of subjects. There can be distinguished two kinds of anonymity:

- *individual* anonymity means that a single respondent is unidentifiable within a given dataset;
- *group* anonymity means that information about a group of respondents cannot be used to violate sensitive features of appropriate distributions.

Methods for providing individual anonymity are discussed in the field of privacy-preserving data publishing (Fung et al. 2010; Wong and Fu 2010). A plenty of methods have been proposed over the years, some of which are randomization (Evfimievski 2002), microaggregation (Domingo-Ferrer and Mateo-Sanz 2002), data swapping (Fienberg and McIntyre 2005), differential privacy (Dwork 2006), etc. A comprehensive overview of recent developments in the field can be found in Sowmyarani and Srinivasan (2012) and Rashid and Yasin (2015).

For the first time, the problem of violating data group anonymity, i.e., anonymity not of individual respondents, but of groups thereof, was introduced in the context of providing group anonymity in Chertov and Tavrov (2010). It was shown that group anonymity can be violated by analyzing outliers of a so called *quantity signal*${\mathbf {q}} = \left( q_1, q_2, \ldots , q_{l_p}\right)$, where each $q_k$, $k = 1,2,\ldots , l_p$, stands for a number of respondents belonging to a given group (e.g., group of military personnel, or group of nuclear scientists) in a given submicrofile, whose total number is $l_p$. A submicrofile is a subset of microfile records sharing the same property, such as region of work. In Chertov and Tavrov (2010), it was argued that outliers in a quantity signal that corresponds to the regional distribution of military personnel can be used to disclose locations of (potentially classified) military bases.

In Chertov and Tavrov (2012), the concept of a quantity signal has been taken further by introducing a *concentration signal*${\mathbf {c}} = \left( c_1, c_2, \ldots , c_{l_p}\right)$, where each $c_k$, $k = 1,2,\ldots , l_p$, is obtained by dividing the corresponding $q_k$ by a total number of records in a corresponding submicrofile. The concentration signal can be used to violate anonymity of groups when absolute numbers of respondents are not sufficient. For instance, as was argued in Chertov and Tavrov (2012) using scientists as an example, extreme ratios of scientists working in a given region could potentially give away the location of a classified research center.

In general, group anonymity can be violated by analyzing such sensitive properties of quantity and concentration signals as (Chertov 2010, p. 77) outliers (almost always a sensitive feature of any distribution), certain statistical features and trends (especially in the case when the quantity signal represents an ordered sequence of numbers), cycles or periods (especially when the quantity signal represents a time series), or frequency spectrum.

In certain practical applications, when the groups are defined in terms of specific attributes (such as a group of military personnel, which is defined by a special attribute uniquely identifying a respondent as a military enlisted), it is possible to protect group anonymity by removing this attribute from the original dataset before publishing. Being a crude solution by itself, it is still not applicable in a number of cases, when it is possible to build an approximation of a group, i.e., define a set of records in the dataset such that its quantity or concentration signal is sufficiently similar to the original one so that it is possible to violate anonymity of the group in question.

Taking into consideration uncertain and imprecise nature of statistical datasets, it was proposed in Chertov and Tavrov (2015) to violate group anonymity with the help of a fuzzy model of a group.

In Chertov and Tavrov (2014), a method for providing group anonymity based on memetic computing was proposed. This method enables us to modify the quantity (or concentration) signal in order to mask its outliers, and at the same time tries to minimize distortion introduced in the dataset. In Tavrov (2015), this algorithm was adapted to work with the fuzzy models proposed in Chertov and Tavrov (2015).

In the next subsection, we will briefly review the concept of fuzzy inference, which is necessary for discussing fuzzy models of groups of respondents.

### Fuzzy inference

The concept of a fuzzy set was first introduced in Zadeh (1965). A *fuzzy setA* in a universal set *X* is a class, in which a point $x\in X$ may have a grade of membership in the interval $\left[ 0, 1\right]$. Each fuzzy set *A* is characterized by a *membership function*$\mu _A: X \rightarrow \left[ 0, 1\right]$, which associates with each $x\in X$ a real number in the interval $\left[ 0, 1\right]$ considered as the “grade of membership” of *x* in *A*.

Fuzzy sets constitute a core of *linguistic variables* (Zadeh 1975). An ordinary *variable* is characterized by a triple $\left( X, U, R\left( X, u\right) \right)$, in which *X* is the name of the variable, *U* is the universe of discourse, *u* is a generic name for the elements of *U*, and $R\left( X, u\right)$ is a subset of *U*, which represents a *restriction* on the values of *u* imposed by *X*. A *fuzzy variable* differs from the ordinary one in that *R* is a *fuzzy* subset of *U*, which represents a *fuzzy restriction* on the values of *u* imposed by *X*.

A linguistic variable differs from an ordinary numerical variable in that its values are not numbers but words or sentences in a natural or artificial language. It is formally characterized by a quintuple $\left( {\mathcal {X}}, T\left( {\mathcal {X}}\right) , U, G, M\right)$, in which ${\mathcal {X}}$ is the name of the variable; $T\left( {\mathcal {X}}\right)$ denotes the term-set of ${\mathcal {X}}$—the set of names of *linguistic values* of ${\mathcal {X}}$, with each value being a fuzzy variable denoted generically by *X* and ranging over a *universe of discourseU*, which is associated with the *base variableu*; *G* is a *syntactic rule* for generating the names, *X*, of values of ${\mathcal {X}}$; and *M* is a *semantic rule* for associating with each *X* its *meaning*, $M\left( X\right)$, which is a fuzzy subset of *U*. The meaning, $M\left( X\right)$, of a term *X* is defined to be the restriction, $R\left( X\right)$, on the base variable *u*, which is imposed by the fuzzy variable named *X*. For example, we can consider a linguistic variable named *Number*, which is associated with the finite term-set $T\left( \text {Number}\right) = \text {few} + \text {several} + \text {many}$, where $+$ denotes union, and in which each term represents a restriction on the values of *u* in the universe of discourse $U = 1 + 2 + \cdots + 10$.

Linguistic variables can be used to formalize knowledge in form of *fuzzy propositions*. While each classical proposition (i.e., a sentence in some language) is required to be either true or false, the truth of fuzzy propositions is a matter of degree. The canonical form of the fuzzy proposition, *p*, is expressed (Klir and Yuan 1995) by the sentence

1 $$\begin{aligned} p: {\mathcal {V}} \text { is } F, \end{aligned}$$

where ${\mathcal {V}}$ is a linguistic variable with the base variable *v* defined on some universal set *V*, and *F* is a fuzzy set on *V* that represents a fuzzy predicate. Given a particular value of *v*, this value belongs to *F* with membership grade $\mu _F\left( v\right)$. This membership grade is then interpreted as the degree of truth, $T\left( p\right)$, of proposition *p*.

Of particular interest for the task of building fuzzy models of groups are conditional propositions (*fuzzy rules*), expressed by the canonical form (Klir and Yuan 1995)

2 $$\begin{aligned} p: \text {If } {\mathcal {X}} \text { is } A,\text { then } {\mathcal {Y}} \text { is } B, \end{aligned}$$

where ${\mathcal {X}}$ and ${\mathcal {Y}}$ are linguistic variables with the base variables *x* and *y* whose values are in sets *X* and *Y*, respectively; *A* and *B* are fuzzy sets on *X* and *Y*, respectively. Antecedents (left parts) of fuzzy rules can contain more than one linguistic variable:

3 $$\begin{aligned} p: \text {If } {\mathcal {X}}_1 \text { is } A_1, \text { and } {\mathcal {X}}_2 \text { is } A_2, \ldots , \text { and } {\mathcal {X}}_n \text { is } A_n,\text { then } {\mathcal {Y}} \text { is } B, \end{aligned}$$

where logical connective *and* can be interpreted as a proper *fuzzy intersection* (Zadeh 1965).

In Chertov and Tavrov (2015), there has been proposed an expert-based procedure for building fuzzy model of a given group to be protected in a form of a fuzzy inference system (Klir and Yuan 1995), i.e., a system which employs expert knowledge in the form of fuzzy rules for making inferences. Such a fuzzy model can be then thought of as a fuzzy classifier that assigns to a given respondent a certain grade of membership in the group.

One of the biggest challenges in creating a fuzzy model of a group is coming up with a comprehensive and complete set of rules. When the number of input variables is relatively big, the total number of consistent fuzzy rules can grow beyond a point when it is all but impossible to use subjective expert knowledge to formalize them.

In some cases, the problem is not only that of defining proper fuzzy rules, but of defining, which variables to account for in the antecedents. For instance, in the case of building a fuzzy model of a group of military personnel, the choice needs to be made as to what microfile attributes need to be considered to make an accurate classification of a given respondent as a military person. In many practical tasks, there is no way of knowing this beforehand, so appropriate efficient search algorithms should be applied, such as evolutionary algorithms.

### Evolutionary approach to building fuzzy rules

*Evolutionary algorithms* are heuristic generate-and-test algorithms that mimic biological evolution by natural selection (Eiben and Smith 2015, p. 5). The task of creating a fuzzy rule set that enables us to violate group anonymity is a complex one, therefore utilizing evolutionary algorithms is a suitable approach to solving this problem.

Historically, application of evolutionary and, in particular, genetic algorithms to evolving rule-based systems was first proposed in Holland (1976) in the context of learning classifier systems. Such systems were described (Eiben and Smith 2015, p. 108) as a framework for studying learning in condition:action rule based systems, using genetic algorithms as the method for the discovery of new rules.

Over the years, evolutionary algorithms have been proposed for evolving fuzzy rules as well. For instance, in Ishibuchi et al. (1995, 1999), there was proposed an evolutionary algorithm for evolving fuzzy classifiers, i.e., rule based systems with fuzzy rules for solving classification tasks. In such systems, consequents (right parts) of the rules in the form (3) are labels of classes of interest rather than linguistic variables.

The task of evolving fuzzy rules for violating group anonymity can be viewed as a task of *subgroup discovery*, which is defined (Wrobel 1997) as the task of finding interesting subgroups in a population of individuals, where interestingness is defined as distributional unusualness with respect to a certain property of interest. Subgroup discovery represents (Jesus et al. 2007) a form of supervised inductive learning, in which, given a set of data and a property of interest to the user, an attempt is made to locate subgroups that are statistically most interesting for the user.

Since the subgroups discovered in data need to be of a more explanatory nature (interpretability of the extracted knowledge for the final user is a crucial aspect), a fuzzy approach (Jesus et al. 2007) for a subgroup discovery process, which considers linguistic variables in descriptive fuzzy rules, is a good approach to take.

It is important to make a distinction between subgroup discovery and the task of classification, because Carmona et al. (2014) subgroup discovery attempts to describe knowledge by data while a classifier attempts to predict the target value for new data to incorporate in the model. In the context of a fuzzy model of a group of respondents, whose anonymity needs to be violated, we are more interested in the classification side. However, many ideas from the field of subgroup discovery can provide useful insight, as will be shown in the paper. An overview of recent developments in the field of subgroup discovery can be found in Atzmueller (2015). Evolutionary algorithms for subgroup discovery are discussed in Carmona et al. (2014).

In general, there can be distinguished two approaches to evolving rule-based systems: Michigan approach (Valenzuela-Rendón 1991) and Pittsburgh approach (Smith 1980). In the first case, each individual in the evolutionary algorithm population corresponds to a single rule. In the second case, each individual is a complete model, i.e., the whole set of rules.

In the extraction of rules for the subgroup discovery task, the Michigan approach is more suited because (Jesus et al. 2007) the objective is to find a reduced set of rules, in which the quality of each rule is evaluated independently of the rest, and it is not necessary to evaluate jointly the set of rules. Moreover, the computation load of the Pittsburgh approach is typically much higher (Ishibuchi et al. 1999, p. 616).

Rules used for describing a subgroup differ in their ability to describe an interesting subgroup, which is measured by a certain quality measure. In general, quality measures can be grouped (Freitas 1999) into objective and subjective measures. Since subjective measures involve experts for evaluating rules, we will focus only on objective measures that are data-driven, and don’t involve expert judgment. A comprehensive overview of quality measures can be found in Lavrač et al. (1999).

However, for the task of violating anonymity of a group of respondents with the help of fuzzy rules in terms of disclosing outliers in the quantity signal, quality measures described in the literature are not suitable. We are interested in cumulative classification properties of fuzzy rules. In other words, we allow ourselves for a certain degree of misclassifications, as long as outliers in the quantity signal obtained with the help of the fuzzy rules correspond to the ones in the original quantity signal. In this work, we propose a novel quality measure that takes this into account.

We also propose a version of an evolutionary algorithm for building a fuzzy model of a group as a set of fuzzy rules, which differs from the ones described in the literature in the quality measure used for evaluating fuzzy rule. The fuzzy model evolved using such an algorithm can be used for violating group anonymity in terms of disclosing outliers in the quantity signal.

## Group anonymity basics

To set a stage for discussing the fuzzy model of a group, we will first introduce some basic notation.

### General group anonymity definitions

Let us define *microdata* as the data about certain respondents presented in a form of a depersonalized *microfile*${\mathbf {M}}$ (i.e., a microfile without identifiers). Each record ${\mathbf {r}}^{\left( i\right) }$, $i = 1,2,\ldots , \rho$, in this microfile contains values of several attributes $w_j$, $j = 1,2,\ldots , \eta$. Let us denote by ${\mathbf {w}}_j$ the set of all the values of $w_j$.

There are two types of attributes of the microfile necessary to define a group. Let $w_{v_j}$, $j = 1,2,\ldots , l$, denote *vital* microfile attributes. These attributes represent those characteristics of records that enable us to determine whether they belong to a group or not. Let us define a *vital value combinationV* as an element of the Cartesian product ${\mathbf {w}}_{v_1} \times {\mathbf {w}}_{v_2} \times \cdots \times {\mathbf {w}}_{v_l}$. Let us denote a set of vital value combinations by ${\mathbf {V}} = \left\{ V_1, \ldots , V_{l_v}\right\}$. We will call records whose attribute values belong to ${\mathbf {V}}$*vital records*. We will denote vital records by ${\mathbf {r}}_v^{\left( i\right) }$, $i = 1,2,\ldots , \rho _v$.

Let $w_p$, $p \ne v_j \forall j$ denote a *parameter* microfile attribute. This attribute determines values, over which we should take the distribution of a group defined by the vital attributes. A *parameter valueP* can be defined as a value of the parameter attribute, i.e., $P \in {\mathbf {w}}_p$. Let us denote a set of parameter values by ${\mathbf {P}} = \left\{ P_1, \ldots , P_{l_p}\right\}$. By their nature, parameter values enable us to divide ${\mathbf {M}}$ into several *submicrofiles*${\mathbf {M}}_1, \ldots , {\mathbf {M}}_{l_p}$. Each submicrofile ${\mathbf {M}}_k$ contains $\rho _k$ records, $k = 1,2,\ldots , l_p$, $\sum _k \rho _k = \rho$. All the records in a certain submicrofile ${\mathbf {M}}_k$ share the same parameter value $P_k$.

A word of caution is in order. Throughout this paper, we will assume that if ${\mathbf {M}}$ contains several attributes that can be concatenated to form a single parameter attribute, they will be concatenated.

We will call all the other attributes $w_{b_j}$, $j = 1,2,\ldots , 1, t$, $b_j \ne p$, $b_j \ne v_i \forall i, j$, *basic attributes*. Obviously, $t = \eta - l - 1$.

The *group* of records $G\left( {\mathbf {V}}, {\mathbf {P}}\right)$, whose distribution needs to be masked when providing group anonymity, can be determined by the values of the vital and parameter attributes. We will denote the distribution of *G*, whose sensitive features need to be protected, by $\Omega \left( {\mathbf {M}}, G\right)$. In consistency with existing literature, we will call this distribution the *goal representation* of a group. Throughout this paper, we will limit ourselves to a particular goal representation most widely used in practice called the *quantity signal*. This signal is denoted by ${\mathbf {q}} = \left( q_1, q_2, \ldots , q_{l_p}\right)$, where each $q_k$, $k = 1,2,\ldots , l_p$, stands for a number of records in ${\mathbf {M}}_k$ that belong to *G*, i.e., whose vital attribute values belong to ${\mathbf {V}}$.

### Quantity signal and its sensitive features

As pointed out before, when providing group anonymity, it is necessary to protect sensitive features of the goal representation under consideration. In this work, we will consider such sensitive features of a quantity signal as its *outliers*. Outliers of a quantity signal might attract attention to parameter submicrofiles that are supposed to be indistinguishable (sites of military bases, classified research centers etc.).

By outliers of a quantity signal, we will understand its values that are statistically inconsistent with the rest of the signal. There have been proposed several approaches to determining outliers in a given dataset. According to the American National Standard of the American Society of Mechanical Engineers ASME PTC 19.1 (ASME 2013, p. 78), two tests are in common usage, the Thompson $\tau$ Technique (Thompson 1935) and the Grubbs Method (Grubbs 1969). In this work, we propose to use the *Modified Thompson*$\tau$*Technique* (MTTT) as the method recommended by ASME (2013, p. 79) for identifying suspected outliers. This method is based on the Student’s *t*-distribution (Student 1908), which is most applicable in situations when the sample size is small, which is typically the case with the quantity signals.

Let the values of the quantity signal ${\mathbf {q}}$ be arranged in increasing order. To determine outliers in this signal, one needs to carry out the following steps:

1. Calculate sample mean and sample standard deviation:
  4 $$\begin{aligned} \overline{{\mathbf {q}}} = \frac{1}{m_{{\mathbf {q}}}}\sum _{i = 1}^{m_{{\mathbf {q}}}}q_i, \quad \sigma _{{\mathbf {q}}} = \sqrt{\frac{\sum _{i=1}^{m_{{\mathbf {q}}}}\left( q_i - \overline{{\mathbf {q}}}\right) ^2}{m_{{\mathbf {q}}} - 1}}, \end{aligned}$$
  where $m_{{\mathbf {q}}}$ is the number of elements in ${\mathbf {q}}$.
2. For each signal value $q_i$, $i = 1,2,\ldots , m_{{\mathbf {q}}}$, calculate absolute value of its deviation from $\sigma _{{\mathbf {q}}}$ as
  5 $$\begin{aligned} d_i = \left| q_i - \overline{{\mathbf {q}}} \right| . \end{aligned}$$
3. Calculate $\tau$ according to
  6 $$\begin{aligned} \tau = \frac{t_{\alpha /2}\cdot \left( m_{{\mathbf {q}}} - 1\right) }{\sqrt{m_{{\mathbf {q}}}}\sqrt{m_{{\mathbf {q}}} - 2 + t_{\alpha /2}^2}}, \end{aligned}$$
  where $t_{\alpha /2}$ is the critical Student’s *t* value (Student 1908) based on significance level $\alpha$ and $m_{{\mathbf {q}}} - 2$ degrees of freedom.
4. If there is such *i* that $d_i > \tau \sigma _{{\mathbf {q}}}$, then $q_i$ is the outlier. In this case, we need to remove $q_i$ from the signal and return to step 1. If $d_i \le \tau \sigma _{{\mathbf {q}}}$ for all *i*, the algorithm stops.

Statistical characteristics (4) are not robust to the presence of outliers in a signal, so there have been proposed (Lanzante 1996) other characteristics:

- the *median*, which can be interpreted as the “middle” value of a signal and is estimated by
  7 $$\begin{aligned} M_{{\mathbf {q}}} = \left\{ \begin{array}{ll} q_{\left( m_{{\mathbf {q}}} + 1\right) /2}, &{}\quad m_{{\mathbf {q}}} \text { is odd}\\ \frac{q_{m_{{\mathbf {q}}}/2} + q_{m_{{\mathbf {q}}}/2 + 1}}{2}, &{}\quad m_{{\mathbf {q}}} \text { is even} \end{array}\right. \end{aligned}$$
- the *pseudo-standard deviation*, which can be defined based on the interquartile range (IQR):
  8 $$\begin{aligned} s_{ps{\mathbf {q}}} = \frac{q_{0.75} - q_{0.25}}{1.349}, \end{aligned}$$
  where $q_{0.75}$ ($q_{0.25}$) is the upper (lower) quartile. If $m_{{\mathbf {q}}}$ is even, the upper (lower) quartile is the median of the largest (smallest) $\frac{m_{{\mathbf {q}}}}{2}$ observations. If the $m_{{\mathbf {q}}}$ is odd, the upper (lower) quartile is the median of the largest (smallest) $\frac{m_{{\mathbf {q}}} + 1}{2}$ observations.

In this work, we will use the MTTT as described above, where estimates (7) and (8) are used in place of estimates (4).

Typically, a set of outliers yielded by MTTT contains signal elements that typically would not be considered as outliers by an expert. Moreover, in some practical cases, not all outliers need to be masked. E.g., when there is a well known military base associated with a particular signal element, masking a corresponding outlier will distort the data and make it obvious that the primary data have been tampered with. Therefore, in the context of providing group anonymity, it is necessary for an expert to revise the set of outliers as determined by MTTT.

Let us denote by $OUT\left( {\mathbf {q}}\right)$ the set of indexes of ${\mathbf {q}}$ that correspond to outliers yielded by MTTT. Let us denote by $OUT_e\left( {\mathbf {q}}\right) \subseteq OUT\left( {\mathbf {q}}\right)$ the subset of indexes of ${\mathbf {q}}$ obtained by excluding from $OUT\left( {\mathbf {q}}\right)$ those indexes, which an expert considers as not important for the task at hand. For brevity, we will also denote by $OUT_e^{\prime }\left( {\mathbf {q}}\right)$ the relative complement of $OUT_e\left( {\mathbf {q}}\right)$ with respect to $\left\{ 1, 2, \ldots , l_p\right\}$.

### The task of providing group anonymity

To solve the *task of providing group anonymity* (TPGA), we need to modify the original microfile ${\mathbf {M}}$ in order obtain a new, protected one ${\mathbf {M}}^{*}$. Such modification needs to meet three conditions (Chertov and Pilipyuk 2011, p. 339):

- disclosure risk is low or at least adequate to importance of information being protected;
- both original and protected microfile data, when analyzed, yield sufficiently similar results;
- the cost of transforming the data is acceptable.

In this paper, by the TPGA, we will understand the task of modifying the microfile in such a way that it is no longer possible to determine outliers in the quantity signal, and at the same time introduce as little distortion as possible in the process.

The easiest “solution” to the TPGA is to recode vital values or remove some of the vital attributes, so that it is impossible to restore the original quantity signal. However, this approach satisfies only one out of three properties stated above, namely, it is easy to carry out. At the same time, this simplistic approach only gives an *impression* of reducing the disclosure risk. As we will demonstrate later, if an adversary has access to appropriate third-party data, sensitive features of the group distribution can be violated under several conditions.

Therefore, even if we choose to remove the vital attributes (or otherwise modify them), we will still need to perform additional microfile modifications in order to properly protect anonymity of a given group.

### Auxiliary microfiles

Let us further on assume that all the vital attributes are removed from ${\mathbf {M}}$. Let us denote by ${\mathbf {M}}^H$ the *harmonized* version of ${\mathbf {M}}$, which can be obtained from by means of two basic transformations:

- attributes $w_{j_1}, \ldots , w_{j_n}$ are replaced by a single *harmonized attribute*$w^H_{j_1}$;
- several values of the $j{\rm {th}}$ attribute $w_j^{(i_1)}, \ldots , w_j^{(i_n)} \in {\mathbf {w}}_j$, $j \in \left. \left\{ v_k\right\} \right| _{k = 1,2,\ldots , l} \cup \left. \left\{ b_k\right\} \right| _{k = 1,2,\ldots , t}$, are replaced by a single value $w_j^{H\left( i_1\right) }$ of the $j{\rm{th}}$ harmonized attribute, which may or may not be equal to any of the values in ${\mathbf {w}}_j$.

Let us denote by $\tilde{{\mathbf {M}}}$ the *auxiliary* microfile with $\tilde{\rho }$ records denoted by $\tilde{{\mathbf {r}}}$, which has the following properties:

- records in $\tilde{{\mathbf {M}}}$ and in ${\mathbf {M}}$ are drawn from sufficiently similar distributions;
- $\tilde{{\mathbf {M}}}$ contains *auxiliary vital attributes* that have the same values and interpretation as the vital attributes in ${\mathbf {M}}$. Auxiliary vital attributes can be used to determine *auxiliary vital records*, whose total number is $\tilde{\rho }_v$. In addition, vital and auxiliary vital records (as well as the records that are not vital or auxiliary vital, respectively) are drawn from sufficiently similar distributions;
- ${\mathbf {M}}$ and $\tilde{{\mathbf {M}}}$ can be transformed into their harmonized versions ${\mathbf {M}}^H$ and $\tilde{{\mathbf {M}}}^H$, so that their basic attributes are identical both in terms of values and their interpretation. More precisely, ${\mathbf {M}}^H$ and $\tilde{{\mathbf {M}}}^H$ contain *harmonized basic* attributes $w^H_{b_j}$, $j = 1,2,\ldots ,t^H$;
- value combinations of attributes $w^H_{b_j}$, $j = 1,2,\ldots ,t^H$, can be used to determine membership grades $\mu _{G}\left( {\mathbf {r}}^{H\left( i\right) }\right)$ of each record ${\mathbf {r}}^{H\left( i\right) } \in {\mathbf {M}}^H$, $i = 1,2,\ldots , \rho$, in a group *G*, whose anonymity needs to be violated;
- an adversary has access to $\tilde{{\mathbf {M}}}$.

It is worth noting that it is not required to harmonize parameter attribute $w_p$ in the original microfile or its analogy $\tilde{w}_p$ in the auxiliary one. Throughout this paper, we will without loss of generality assume that $w_p$ and $\tilde{w}_p$ remain intact during the harmonization process.

If the conditions given above are met, it is possible to build a set of fuzzy rules to determine membership grades $\mu _{G}\left( {\mathbf {r}}^{H\left( i\right) }\right)$, $i = 1,2,\ldots , \rho$, of each record in a group. This set of rules can be interpreted as a *fuzzy model* of the group whose anonymity needs to be violated. This model enables us to construct an *auxiliary quantity signal*${\mathbf {q}}^{aux} = \left( q^{aux}_1, \ldots , q^{aux}_{l_p}\right)$, where $q^{aux}_j$, $j = 1, 2, \ldots , l_p$, are defined by

9 $$\begin{aligned} q^{aux}_j = \sum _{{\mathbf {r}}^H\in {\mathbf {M}}_j^H | \mu _G\left( {\mathbf {r}}^H\right) \ge \alpha } \mu _{G}\left( {\mathbf {r}}^H\right) , \end{aligned}$$

where ${\mathbf {M}}_j^H$ is the parameter submicrofile of ${\mathbf {M}}^H$, whose records share the same parameter value $P_j$; $\alpha$ is the *group membership threshold* used to cut off records that don’t belong to *G* with a sufficiently high grade. Throughout this paper, we will use $\alpha = 0.5$.

The auxiliary quantity signal ${\mathbf {q}}^{aux}$ doesn’t have to be close in a numerical sense to the original quantity signal ${\mathbf {q}}$—it is only required that outliers in ${\mathbf {q}}^{aux}$ correspond to those ones in ${\mathbf {q}}$.

### Fuzzy rules in a fuzzy model of a group

In order to construct the auxiliary quantity signal as defined by (9), we need to calculate membership grades $\mu _{G}\left( {\mathbf {r}}^{H\left( i\right) }\right)$ of each microfile record ${\mathbf {r}}^{H\left( i\right) } \in {\mathbf {M}}^H$, $i = 1,2,\ldots ,\rho$. In general, this can be done using appropriate fuzzy rules.

For the case of a fuzzy model of a group, such fuzzy rules can be presented in the following form:

10 $$\begin{aligned} R_i: \text {If } L_1 \text { is } A_{i1}, \text { and } L_2 \text { is } A_{i2} , \ldots ,\text { and } L_{t^H} \text { is } A_{it^H} \text { then } G, \end{aligned}$$

where $R_i$, $i = 1, 2, \ldots , m$, denotes the $i\hbox {th}$ fuzzy rule, $A_{ij}$ denotes the value of the $j\hbox {th}$ linguistic variable $L_j$ used in the $i\hbox {th}$ fuzzy rule, *G* denotes the class of records that belong to a group.

Each linguistic variable $L_j$ in the fuzzy rules, $j = 1, 2, \ldots , t^H$, corresponds to the attribute $w^H_{b_j}$, $j = 1,2,\ldots , t^H$, in the harmonized microfile ($\tilde{{\mathbf {M}}}^H$ or ${\mathbf {M}}^H$). It has several values $LL_j^k$, $k = 1, 2, \ldots , l_{L_j}$, with their membership functions denoted by $\mu _{LL_j^k}$. In addition, each linguistic variable by default has a value $LL_j^0$ with the membership function $\mu _{LL_j^0} \equiv 1$. If $A_{ij} = LL_j^0$ is present in a fuzzy rule $R_i$, it means that the actual value of attribute $w^H_{b_j}$ is discarded. As pointed out in Ishibuchi et al. (1999), in this way we can obtain fuzzy rules of different generalization capacity.

For each linguistic variable, we can define a range $\left[ l\left( L_j\right) , u\left( L_j\right) \right]$ of acceptable values of a corresponding base variable. All the records from ${\mathbf {M}}^H$ and $\tilde{{\mathbf {M}}}^H$, whose values of attributes $w^H_{b_j}$ lie outside the specified ranges, $j = 1, 2, \ldots , t^H$, need to be removed. In order not to complicate the notation, we will further on assume that ${\mathbf {M}}^H$ and $\tilde{{\mathbf {M}}}^H$ denote microfiles that contain only those records, whose attribute values lie inside corresponding ranges, unless specified otherwise. Similarly, we will further on assume that values $\rho$ and $\tilde{\rho }$ denote the total number of records in ${\mathbf {M}}^H$ and $\tilde{{\mathbf {M}}}^H$, respectively, where ${\mathbf {M}}^H$ and $\tilde{{\mathbf {M}}}^H$ denote either original microfiles or microfiles with records whose attribute values belong to specified ranges, depending on the context.

In what follows, we will make use of notation accepted in the subgroup discovery field. Let us define the *antecedent part compatibility* (Jesus et al. 2007) as the degree of compatibility between a record ${\mathbf {r}}$ and the antecedent part of $R_i$ as

11 $$\begin{aligned} APC\left( {\mathbf {r}}, R_i\right) = \prod _j \mu _{A_{ij}}\left( r_{b_j}\right) , \end{aligned}$$

where $\mu _{A_{ij}}$ is the membership function of the fuzzy set $A_{ij}$, $\prod$ denotes a proper fuzzy intersection. Throughout this paper, we will use arithmetic product as the fuzzy intersection.

To account for the group membership threshold *alpha* introduced in (9), we will further on use the following modification of (11):

12 $$\begin{aligned} APC^{\alpha }\left( {\mathbf {r}}, R_i\right) = \left\{ \begin{array}{ll} APC\left( {\mathbf {r}}, R_i\right) , &{}\quad APC\left( {\mathbf {r}}, R_i\right) \ge \alpha \\ 0, &{}\quad \text {otherwise} \end{array} \right. . \end{aligned}$$

Then, we can say that

$$\begin{aligned} \mu _{G}\left( {\mathbf {r}}\right) = \bigcup _{i = 1}^m APC^{\alpha }\left( {\mathbf {r}}, R_i\right) , \end{aligned}$$

where $\bigcup$ denotes fuzzy union (Zadeh 1965). In this work, we will use maximum function as the fuzzy union.

We say that a record ${\mathbf {r}}$*verifies* the antecedent part of $R_i$ if $APC^{\alpha }\left( {\mathbf {r}}, R_i\right) > 0$, and that it is *covered* by $R_i$ if additionally ${\mathbf {r}} \in G$.

In the context of violating group anonymity in terms of disclosing outliers in the auxiliary quantity signal, we are interesting in cumulative classification properties of the fuzzy rules. In other words, we allow ourselves for a certain degree of misclassifications, as long as outliers in the auxiliary quantity signal obtained with the help of the fuzzy rules correspond to the ones in the original quantity signal.

Therefore, we need to introduce quality measures that are different from the ones described in the literature:

- a fuzzy rule should have reasonable *discriminative capability*:
  13 $$\begin{aligned} \frac{\sum _{\tilde{{\mathbf {r}}}\in G} APC^{\alpha }\left( \tilde{{\mathbf {r}}}, R_i\right) }{\tilde{\rho }_v} > \frac{\sum _{\tilde{{\mathbf {r}}}\in \tilde{{\mathbf {M}}}^H} APC^{\alpha }\left( \tilde{{\mathbf {r}}}, R_i\right) }{\tilde{\rho }}, \end{aligned}$$
  which means that rule $R_i$ classifies as belonging to the group *G* a disproportionally bigger number of auxiliary vital records than auxiliary records in general. We will introduce a *discriminative factor* defined by
  14 $$\begin{aligned} DF\left( R_i\right) = \frac{\sum _{\tilde{{\mathbf {r}}}\in G} APC^{\alpha }\left( \tilde{{\mathbf {r}}}, R_i\right) }{\tilde{\rho }_v} - \frac{\sum _{\tilde{{\mathbf {r}}}\in \tilde{{\mathbf {M}}}^H} APC^{\alpha }\left( \tilde{{\mathbf {r}}}, R_i\right) }{\tilde{\rho }}, \end{aligned}$$
- a fuzzy rule should have reasonable *relative confidence*:
  15 $$\begin{aligned} \frac{\sum _{\tilde{{\mathbf {r}}}\in G} APC^{\alpha }\left( \tilde{{\mathbf {r}}}, R_i\right) }{\sum _{\tilde{{\mathbf {r}}}\in \overline{G}} APC^{\alpha }\left( \tilde{{\mathbf {r}}}, R_i\right) } \ge \gamma , \end{aligned}$$
  which means that $R_i$*incorrectly* classifies no more than $\frac{\sum _{\tilde{{\mathbf {r}}}\in G} APC^{\alpha }\left( \tilde{{\mathbf {r}}}, R_i\right) }{\gamma }$ records as belonging to *G*, where $\gamma$ will be called the *relative confidence threshold*. We will introduce the *relative confidence factor* defined by
  16 $$\begin{aligned} RCF\left( R_i\right) = \frac{\sum _{\tilde{{\mathbf {r}}}\in G} APC^{\alpha }\left( \tilde{{\mathbf {r}}}, R_i\right) }{\sum _{\tilde{{\mathbf {r}}}\in \overline{G}} APC^{\alpha }\left( \tilde{{\mathbf {r}}}, R_i\right) }. \end{aligned}$$

It can be recognized that the minuend from (14) is a fuzzy version of a well-known quality measure called *support*, and the subtrahend is a fuzzy version of another quality measure called *coverage* (Lavrač et al. 2004). Support considers the number of examples satisfying both the antecedent and the consequent parts of the rule, whereas coverage measures the percentage of examples covered on average by one rule.

It can also be recognized that (16) resembles the quality measure called *confidence* introduced in Jesus et al. (2007). However, our version differs in the denominator. Classically, the division is performed over the sum of the degree of membership of *all* the records that verify the antecedent part of this rule, whereas in our version we consider only those records that verify the antecedent part of the rule and *don’t belong toG*. In our view, this makes interpretation of this quality measure more tractable, because it can be easily assessed how many respondents the rule classifies incorrectly, in relative terms.

In a fuzzy model of a group, each rule $R_i$ needs to have quality measures with the following properties: $DF\left( R_i\right) > 0$, $RCF\left( R_i\right) \ge \gamma$. In this case, we will reduce misclassifications, and thereby obtain a more suitable auxiliary quantity signal.

Auxiliary quantity signal contains all the information necessary to violate group anonymity. On the other hand, to protect group anonymity, we need to use a signal that consists of crisp values representing numbers of respondents, not fuzzy degrees. Let us introduce a *crisp auxiliary quantity signal*:

17 $$\begin{aligned} q^{aux}_{\text {crisp}_j} = \left| {\mathbf {r}}^H\in {\mathbf {M}}_j^H | \mu _G\left( {\mathbf {r}}^H\right) \ge \alpha \right| . \end{aligned}$$

Values of (17) correspond to quantities of records in a corresponding microfile, which are assigned a membership grade greater than $\alpha$. We will make use of the signal defined in this way when we will discuss the method for protecting group anonymity in one of the subsequent sections.

As it was mentioned earlier, due to complicated interrelations between different rules in the rule base, it is virtually impossible to construct the rule base from scratch using only expert knowledge. In sections to follow, we will present an appropriately tailored evolutionary algorithm for solving this task.

### Adequacy of the fuzzy model of a group

In this section, we will briefly discuss possible tests for evaluating adequacy of the fuzzy model of the group described above. By adequacy of the fuzzy model we will consider its ability to correctly determine outliers in the quantity signal, i.e., how similar are the outliers in the original and auxiliary quantity signals. It therefore seems natural to evaluate model adequacy using tests designed to evaluate accuracy of classifiers.

Let $X = {\mathbb {R}}^n$ be the multidimensional pattern space under investigation, each element ${\mathbf {x}}\in X$ of which belongs to one of the two classes from the set $Y = \left\{ C_1, C_2\right\}$. Let $P_{XY}$ be the unknown joint distribution over $X \times Y$. Let us be given a classifier $f: X \rightarrow Y$ that maps each pattern ${\mathbf {x}}\in X$ to a certain class. Let $\epsilon = E_{XY}\left[ f\left( x\right) \ne y\right]$ be the classifier error, where *E* is the expectation operator.

Since in practical cases *X* is typically a set of finite size, $\epsilon$ can only be estimated. Let $S = \left\{ \left( {\mathbf {x}}_1, y_1\right) , \ldots , \left( {\mathbf {x}}_m, y_m,\right) \right\}$ be the set of pairs drawn from $P_{XY}$. Let us introduce the *confusion matrix* (Olivetti et al. 2012)

18 $$\begin{aligned} Z = \left( \begin{array}{ll} TP &{} FP\\ FN &{} TN \end{array}\right) , \end{aligned}$$

where *TP* (true positive) is the number of patterns from *S* that belong to class $C_1$, and for which $f\left( {\mathbf {x}}\right) = C_1$; *FP* (false positive) is the number of patterns from *S* that belong to class $C_1$, and for which $f\left( {\mathbf {x}}\right) = C_2$; *FN* (false negative) is the number of patterns from *S* that belong to class $C_2$, and for which $f\left( {\mathbf {x}}\right) = C_1$; *TN* (true negative) is the number of patterns from *S* that belong to class $C_2$, and for which $f\left( {\mathbf {x}}\right) = C_2$.

The sum of values of (18) is *m*. Let us denote by *e* the number of incorrectly classified patterns. Then, $\sum _i Z_{ii} = m - e$.

The *prediction accuracy* is defined as

19 $$\begin{aligned} PA = \frac{m - e}{m}. \end{aligned}$$

When the number of patterns per class is not equal, a setting is called unbalanced. As was shown in Olivetti et al. (2012), test (19) is not suitable for unbalanced data. One of the tests suitable for unbalanced data is Youden’s *J* statistic (Youden 1950):

20 $$\begin{aligned} J = \frac{TP}{TP + FN} + \frac{TN}{FP + TN} - 1. \end{aligned}$$

This test explicitly captures the type I and type II errors.

In Olivetti et al. (2015), there was proposed a Bayesian test of statistical independence between the results given by the classifier, on the one hand, and the true distribution $P_{XY}$, on the other hand. This test also takes into account the unbalanced nature of the data and the size of the data set. Let us denote by $H_0$ the hypothesis that the results given by the classifier are statistically independent of the true distribution $P_{XY}$. Let us also denote by $H_1$ the hypothesis that such results are statistically dependent. Then, let us denote by *B* the *Bayes factor* that measures the evidence of the data in favor of $H_1$ with respect to $H_0$:

21 $$\begin{aligned} B\left( t_1, t_2\right)= & {} \left[ \frac{TP + FP + FN + TN + 1}{\left( TP + FP + t_1 + 1\right) \left( FN + TN + t_2 + 1\right) } \right] \nonumber \\&\times \,\left[ \frac{\left( t_1 + 1\right) \left( t_2 + 1\right) }{t_1 + t_2 + 1}\right] \cdot \left( \begin{array}{c}TP + FP + FN + TN\\ TP + FN\end{array}\right) \nonumber \\&\times \, \sum _{i = 0}^{t_1} \sum _{j = 0}^{t_2}\frac{\left( \begin{array}{c}t_1\\ i\end{array}\right) ^2 \left( \begin{array}{c}t_2\\ j\end{array}\right) ^2}{\left( \begin{array}{c}t_1 + t_2\\ i + j\end{array}\right) \left( \begin{array}{c}TP + FP + t_1\\ TP + i\end{array}\right) \left( \begin{array}{c}FN + TN + t_2\\ FN + j\end{array}\right) }, \end{aligned}$$

where $$\left( \begin{array}{c}i\\ j\end{array}\right) = \frac{i!}{j!\left( i - j\right) !}$$; $t_1$ and $t_2$ are non-negative integer parameters.

The test for evaluating the classifier based on (21) is calculated by

22 $$\begin{aligned} MB = {\mathop {\mathop {min}\limits _{0\le t_1\le m}}\limits _{0\le t_2\le m}}\log B\left( t_1, t_2\right) . \end{aligned}$$

Guidelines for the interpretation of this test are given in Table 1 (Kass and Raftery 1995).

**Table 1:** Guidelines for the interpretation of *MB* in terms of the strength of evidence in favor of $H_1$ against $H_0$

*MB*	$<$0	0–1	1–3	3–5	$>$5
$H_1$ strength	Negative	Bare mention	Positive	Strong	Decisive

In the context of evaluating the adequacy of the fuzzy model of a given group, the pattern space has to be taken as a set of parameter values: $X = {\mathbf {P}}$. Class $C_1$ contains those parameter values that correspond to outliers in ${\mathbf {q}}$, $C_2$ contains all the other parameter values.

The auxiliary quantity signal ${\mathbf {q}}^{aux}$ can differ from ${\mathbf {q}}$ in two ways:

- some of the outliers in ${\mathbf {q}}$ don’t have a correspondence in ${\mathbf {q}}^{aux}$, i.e., we cannot violate anonymity of some of the outliers (type II errors). We will call such outliers *undisclosed outliers*;
- some of the outliers in ${\mathbf {q}}^{aux}$ don’t have a correspondence in ${\mathbf {q}}$, i.e., the fuzzy rules introduce additional outliers not supported by real data (type I errors). We will call such outliers *false outliers*.

Taking into consideration notation introduced earlier, elements of the confusion matrix (18) can be defined as follows:

- $$TP = \left| OUT_e\left( {\mathbf {q}}\right) \cap OUT_e\left( {\mathbf {q}}^{aux}\right) \right|$$;
- $$FP = \left| OUT_e\left( {\mathbf {q}}\right) \cap OUT_e^{\prime }\left( {\mathbf {q}}^{aux}\right) \right|$$;
- $$FN = \left| OUT_e^{\prime }\left( {\mathbf {q}}\right) \cap OUT_e\left( {\mathbf {q}}^{aux}\right) \right|$$;
- $$TN = \left| OUT_e^{\prime }\left( {\mathbf {q}}\right) \cap OUT_e^{\prime }\left( {\mathbf {q}}^{aux}\right) \right|$$.

### General approach to applying fuzzy rules to violating group anonymity

In general, to violate anonymity of a certain group *G* in a microfile ${\mathbf {M}}$ in terms of disclosing outliers in its quantity signal, we need to proceed along the following steps:

1. *Harmonization* Choose a microfile ${\mathbf {M}}$ and determine a group *G* of records, whose distribution should be disclosed. Choose an auxiliary microfile $\tilde{{\mathbf {M}}}$ that satisfies all the conditions given earlier. Perform harmonization of ${\mathbf {M}}$ and $\tilde{{\mathbf {M}}}$ and obtain harmonized microfiles ${\mathbf {M}}^H$ and $\tilde{{\mathbf {M}}}^H$ that have identical attributes with two exceptions: parameter attributes in both harmonized microfiles may not be identical, and $\tilde{{\mathbf {M}}}^H$ contains auxiliary vital attributes, whereas ${\mathbf {M}}^H$ has vital attributes removed.
2. *Input Variables Identification* For each linguistic variable $L_j$ corresponding to a basic harmonized attribute $w^H_{b_j}$, $j = 1,2,\ldots , t^H$, define a range of values of its base variable $\left[ l\left( L_j\right) , u\left( L_j\right) \right]$. Remove from ${\mathbf {M}}^H$ and $\tilde{{\mathbf {M}}}^H$ records whose values of attributes $w^H_{b_j}$ lie outside the specified ranges, $j = 1,2, \ldots , t^H$. Use expert judgment to determine the fuzzy values $LL_j^k$ for each linguistic variable $L_j$, $j = 1,2,\ldots , t^H$, $k = 1,2, \ldots , l_{L_j}$, defined by appropriate membership functions denoted by $\mu _{LL_j^k}$.
3. *Evolution* Use the evolutionary algorithm to evolve fuzzy rules for violating anonymity of *G* in ${\mathbf {M}}^H$ based on the data from $\tilde{{\mathbf {M}}}^H$. To reduce the number of undisclosed and false outliers, select only those rules *R*, for which $DF\left( R\right) > 0$ and $RCF\left( R\right) \ge \gamma$, and whose support is greater than a predefined value $\kappa$. To reduce computational overhead, remove rules that are more specific versions of other rules in the set, i.e., for each pair of rules $R_i$ and $R_j$, if $\forall k \quad A_{ik} \ne A_{jk} \rightarrow A_{ik} = LL_k^0$, remove $A_j$. Using the fuzzy rules obtained, assign membership grades to all the records in ${\mathbf {M}}^H$, uniting the results in the fuzzy sense.
4. *Disclosing Outliers* Construct the auxiliary quantity signal (9) and determine outliers in it.

## Evolutionary algorithm for building the fuzzy model of a group

### Outline of the evolutionary algorithm

In the proposed algorithm, whose outline corresponds to the outline presented in Ishibuchi et al. (1995), we perform evolution only at the level of fuzzy rules. This means that we do not perform any fine-tuning of membership functions of input variables. We choose this approach to preserve comprehensibility for humans of the fuzzy rules in the system.

The outline of the algorithm is as follows:

1. Randomly generate initial population ${\mathbf {R}} = \left\{ R_i \right\}$ of $\mu$ individuals, $i = 1,2,\ldots , \mu$.
2. Calculate values of the fitness function for each individual: $f\left( R_i\right)$, $i = 1,2,\ldots , \mu$.
3. Check termination condition: if it is satisfied, stop; continue otherwise.
4. Select $\lambda$ pairs of individuals and put them into set ${\mathbf {R}}^{\prime }$.
5. Recombine pairs of individuals from ${\mathbf {R}}^{\prime }$ with a *recombination operator*$REC\left( R_i, R_j\right)$, $i = 1,2,\ldots , \lambda$, $j = \lambda + 1, \ldots , 2\cdot \lambda$. Put the offspring into set ${\mathbf {R}}^{\prime \prime }$.
6. Mutate individuals from ${\mathbf {R}}^{\prime \prime }$ with a *mutation operator*$MUT\left( R_j\right)$, $j = 1,2,\ldots , \lambda$.
7. Replace $\lambda$ individuals from ${\mathbf {R}}$ that have the lowest fitness values with the mutated offspring.
8. Go to step 3.

### Representation and fitness function

In this work, we treat each individual $R_i \in {\mathbf {R}}$, $i = 1,2,\ldots , \mu$, as a single rule in the fuzzy rule set being evolved. I.e., the whole population constitutes the whole fuzzy rule set, in full concordance with the Michigan approach.

We propose to represent each rule $R_i$, $i = 1,2,\ldots , \mu$, as a vector of integer values

23 $$\begin{aligned} R_i = \left( R_{i1}, R_{i2}, \ldots , R_{it^H}\right) , \end{aligned}$$

where $R_{ij}$ is a certain index of the fuzzy value of a linguistic variable $L_j$.

Availability of values $LL_j^0$, $j = 1,2,\ldots , t^H$, in $R_i$ enables us to evolve rules that don’t take into account values of the attribute $w^H_{b_j}$. In other words, the evolutionary process can lead to obtaining more generalized rules.

In this work, we evaluate fitness of each individual $R_i$ in terms of its quality measures introduced earlier:

24 $$\begin{aligned} f\left( R_i\right) = \left\{ \begin{array}{ll} DF\left( R_i\right) \cdot RCF\left( R_i\right) ,&{} \quad DF\left( R_i\right) > 0\\ 0,&{} \quad DF\left( R_i\right) \le 0 \end{array} \right. , \quad i = 1,2,\ldots ,\mu . \end{aligned}$$

### Other algorithm parameters

Operator $REC\left( R_{i_1}, R_{i_2}\right)$ should be a proper recombination operator for integer representation applied with a high probability $p_c$ to two individuals $R_{i_1}$ and $R_{i_2}$ that yields two offspring individuals $R_{j_1}$ and $R_{j_2}$. Operator $MUT\left( R\right)$ should be a proper mutation operator for integer representation applied with a low probability $p_m$ to a single individual *R* that yields the mutated one $R^{\prime }$.

In this paper, we will use *uniform crossover* (Syswerda 1989) as a recombination operator and *random resetting mutation* (Eiben and Smith 2015, p. 43) as a mutation operator. We will also choose the following algorithm parameters:

- we will choose tournament selection (Brindle 1981) as an efficient and easy to implement selection operator, with the tournament size 10;
- we will create initial populations by randomly generating values of each fuzzy rule element $R_{ij}$, $i = 1,2,\ldots , \mu$, $j = 1,2,\ldots , t^H$, from a uniform distribution on $\left[ 0, l_{L_j}\right]$;
- we will choose the number of generations *N* as a termination condition, i.e., we will terminate the algorithm after having obtained *N* consequent populations.

## Memetic algorithm for protecting group distributions

### General information

In previous sections, we have shown that the TPGA is a pressing one, and group distributions need to be protected even when vital attributes are removed from the microfile. In this section, we will discuss the *memetic algorithm* (MA) for solving the task of providing group anonymity. This algorithm was introduced in Chertov and Tavrov (2014), and we will heavily rely on that publication when presenting the algorithm here.

We will assume that the data publisher decides to remove vital attributes from the microfile. As pointed out before, to provide group anonymity, we need to mask outliers in an auxiliary quantity signal obtained using appropriate fuzzy rules.

The general outline of a single-stage approach to solving the TPGA is as follows:

1. Prepare a (depersonalized) microfile ${\mathbf {M}}$ representing data to be anonymized.
2. Define groups of respondents $G_i\left( {\mathbf {V}}_i, {\mathbf {P}}_i\right)$, whose quantity signals need to be masked, $i = 1,2,\ldots , k$.
3. For each *i* from 1 to *k*:
  - Build the quantity signal ${\mathbf {q}}_i$ for $G_i$.
  - Obtain fuzzy models of $G_i$ using the evolutionary algorithm.
  - Build the auxiliary quantity signal ${\mathbf {q}}^{aux}_i$ for $G_i$ using the obtained fuzzy models, and the corresponding crisp auxiliary quantity signal ${\mathbf {q}}^{aux}_{\text {crisp}_i}$.
  - Compare two signals and determine whether there is risk of violating group anonymity in terms of disclosing their outliers.
  - If there is such risk, define the *modifying transformation*$A: {\mathbf {q}}^{aux}_{\text {crisp}_i} \left( {\mathbf {M}}, G_i\right) \rightarrow {\mathbf {q}}^{aux*}_{\text {crisp}_i}\left( {\mathbf {M}}^{*}, G_i\right)$, obtain the *modified crisp auxiliary quantity signal*${\mathbf {q}}^{aux*}_{\text {crisp}_i}$, and hence the *modified microfile*${\mathbf {M}}^{*}$.
4. Prepare the modified microfile ${\mathbf {M}}^{*}$ for publishing.

In order to modify the auxiliary quantity signal for a given group in a given microfile, we need to physically alter some of the values in the microfile, more precisely, alter parameter values for certain records. To preserve the number of records with a particular parameter value, the records have to be altered in pairs, which can be interpreted as swapping the records between submicrofiles. One record needs to belong to the fuzzy model of a group, and another needs not to.

As mentioned before, to solve the TPGA means not only to modify the auxiliary quantity signal, but also to introduce as little distortion into the microfile as possible. To this end, the records being swapped have to be close to each other is some sense. In this work, we will apply the *influential metric* (Chertov 2010) to determine the degree of similarity between two microfile records. This metric is defined in terms of so called *influential attributes*, i.e., those ones whose distribution is important for further researches using microfile data. In this work, we will assume that influential attributes are the same as the basic harmonized attributes.

The influential metric is defined as

25 $$\begin{aligned} \text {InfM}({\mathbf {r}}, {\mathbf {r}}^{*}) = \sum _{p = 1}^{n_{ord}}\omega _p\left( \frac{r_{I_p} - r^{*}_{I_p}}{r_{I_p} + r^{*}_{I_p}}\right) ^2 + \sum _{k = 1}^{n_{cat}}\gamma _k\chi ^2\left( r_{J_k}, r^{*}_{J_k}\right) , \end{aligned}$$

where $I_p$ is the $p\hbox {th}$ ordinal basic attribute (their overall number is $n_{ord}$), $J_k$ is the $k\hbox {th}$ categorical basic attribute (their overall number is $n_{cat}$), $\chi \left( v_1, v_2\right)$ denotes the operator that equals to $\chi _1$ if values $v_1$ and $v_2$ fall into one category, and equals to $\chi _2$ otherwise, $\omega _p$ and $\gamma _k$ are non-negative weighting coefficients (the bigger the coefficient, the more important is the attribute for the researches).

Preserving data utility from the minimal data distortion point of view is a task of high complexity and dimensionality, therefore, it is a good idea to use MAs (Moscato 1989) to solve the TPGA. MAs are typically implemented as evolutionary algorithms with local search procedures (Eiben and Smith 2015, p. 173). New applications of MAs to solving complex optimization tasks can be found in Kumar et al. (2014).

### Outline of the algorithm

An outline of a memetic algorithm for modifying the microfile ${\mathbf {M}}$ in order to protect outliers in corresponding quantity signal is as follows:

1. Create population *P* of $\mu$ individuals, apply to them *local search operatorS*.
2. Calculate fitness function $f\left( {\mathbf {x}}\right)$ for each individual ${\mathbf {x}} \in P$.
3. Check termination condition. It if holds, stop, otherwise, go to 4.
4. Select $\lambda$ pairs of parents.
5. Apply *recombination operatorR* to each parent pair.
6. Apply *mutation operatorM* to each of offspring. Put the offspring into $P^{\prime }$.
7. Apply local search operator *S* to each individual ${\mathbf {x}} \in P^{\prime }$.
8. Calculate fitness function $f\left( {\mathbf {x}}\right)$ for each individual ${\mathbf {x}} \in P^{\prime }$.
9. Select $\mu$ individuals from $P \cup P^{\prime }$, put them into *P* in place of current ones.
10. Go to 3.

In the algorithm outline above, we made use of several symbols introduced earlier, but with a different meaning. We hope it will be understandable from the context, what symbols mean in each particular case.

Each individual is a matrix *U* with *Q* rows and four columns with the following elements:

1. The first column contains indexes $u_{i1}\; \forall i = 1,2,\ldots , Q$ of submicrofiles to remove vital records from. The user has to define the set of such submicrofiles.
2. The third column contains indexes $u_{i3} \; \forall i = 1,2,\ldots , Q$ of submicrofiles to add vital records to. The user has to define the set of such submicrofiles.
3. The second column contains indexes $u_{i2} \; \forall i = 1,2,\ldots , Q$ of the records from ${\mathbf {M}}_{u_{i1}}$ to be removed.
4. The fourth column contains indexes $u_{i4} \; \forall i = 1,2,\ldots , Q$ of the records from ${\mathbf {M}}_{u_{i3}}$ to be swapped with the ones defined by $u_{i2}$.

By its nature, each individual *U* uniquely defines the modified quantity signal ${\mathbf {q}}^{*}$, and also determines the particular way of obtaining it, because each row in *U* defines a particular pair of respondents to be swapped. Thereby, each *U* defines a complete solution to the TPGA at hand.

Two restrictions are imposed on each individual *U*:

- a submicrofile index *i* can occur in the first column of *U* not more than $q_i$ times;
- each pair $\langle u_{i1}, u_{i2}\rangle$ or $\langle u_{i3}, u_{i4}\rangle \; \forall i=1,2,\ldots , Q$ cannot occur in *U* more than once.

These restrictions cannot be violated throughout the algorithm run.

In this work, we propose to use the fitness function as the product

26 $$\begin{aligned} f\left( U\right) = \Upsilon \left( U\right) \cdot \Phi \left( U\right) \cdot \Psi \left( U\right) , \end{aligned}$$

where $\Upsilon \left( U\right)$ gives estimation of the solution quality in terms of minimizing microfile distortion, $\Phi \left( U\right)$ gives estimation the solution quality in terms of protecting outliers in the quantity signal, and $\Psi \left( U\right)$ is a penalty term against obtaining individuals with too many rows.

We propose to use the following expression for the first term of (26):

27 $$\begin{aligned} \Upsilon \left( U\right) = \frac{C_{\max } - \sum \nolimits _{i = 1}^Q\text {InfM}\left( {\mathbf {M}}_{u_{i1}}\left( u_{i2}\right) , {\mathbf {M}}_{u_{i3}}\left( u_{i4}\right) \right) }{C_{\max }}, \end{aligned}$$

where $C_{\max }$ is the greatest possible value of the cumulative influential metric (25), ${\mathbf {M}}_i\left( j\right)$ is the operator yielding the $j\hbox {th}$ record of the submicrofile ${\mathbf {M}}_i$, $i = 1,2,\ldots , l_p$.

Other terms of the fitness function can be chosen depending on the TPGA at hand.

In this work, we use the following recombination operator $R\left( U_{i_1}, U_{i_2}\right)$. It generates two random *crossover points*$k_1\in \left[ 0, Q_{i_1}\right]$ and $k_2\in \left[ 0, Q_{i_2}\right]$, splits each parent at appropriate points, exchanges the tails between them, and thus creates the offspring. This operator has to be applied with a high probability $p_c$.

We also use the mutation operator that is a superposition $M = M_4\circ M_3\circ M_2\circ M_1$ of the following operators:

1. $M_1$ is a *swap mutation* operator (Syswerda 1991) applied with a small probability $p_{m_1}$ to the first column of *U*. Each pair $\left\langle u_{i1}, u_{i2} \right\rangle$ needs to be preserved $\forall i = 1,2,\ldots , Q$.
2. $M_2$ is also a swap mutation operator applied with a small probability $p_{m_2}$ to the third column of *U*. Each pair $\left\langle u_{i3}, u_{i4} \right\rangle$ needs to be preserved $\forall i = 1,2,\ldots , Q$.
3. $M_3$ is a random resetting mutation operator (Eiben and Smith 2015, p. 43) applied with a small probability $p_{m_3}$ to the second column of *U*.
4. $M_4$ is a random resetting mutation operator applied with a small probability $p_{m_4}$ to the fourth column of *U*.

In this work, we use the following local search memetic operator $S\left( U\right)$:

1. Carry out steps 2–4 $\forall i = 1,2,\ldots , Q$.
2. Generate a uniformly distributed number $r\in \left[ 0, 1\right]$.
3. If $r \le p_{mem}$, assign to $u_{i4}$ the index of a record from ${\mathbf {M}}_{u_{i3}}$ closest to the record defined by $u_{i2}$ from ${\mathbf {M}}_{u_{i1}}$ in terms of (25). Otherwise, assign to $u_{i2}$ the index of a record from ${\mathbf {M}}_{u_{i1}}$ closest to the record defined by $u_{i4}$ from ${\mathbf {M}}_{u_{i3}}$ in terms of (25).
4. Go to step 2.

Other MA components, such as selection, initialization, termination, population size etc. should be chosen individually for each TPGA to be solved.

## Results

### Problem definition and microfile harmonization

To illustrate ideas developed in this work, we decided to set a task of violating anonymity of a group of regionally distributed military personnel in the U.S. Outliers in quantity signals representing such a distribution might point to sites of military facilities, some of which might potentially be classified.

We decided to choose the 1 % sample microfile of the American Community Survey (ACS) conducted in 2013 available from the IPUMS-International Project (Ruggles et al. 2010) as the microfile ${\mathbf {M}}$ we would like to violate group anonymity in. This microfile contains $\rho = 1{,}380{,}924$ records.

The microfile contains attributes *Place of work: state, 1980 onward* and *Place of work: PUMA, 2000 onward* (where PUMA stands for Public Use Microdata Area), that, if concatenated, give a unique code of a PUMA where a respondent works. We decided to replace these attributes with a single one called *Place of work* by concatenating the values of the attributes for each microfile record. The newly obtained attribute plays the role of the parameter attribute for our task.

The microfile also contains $l = 1$ vital attribute *Occupation, SOC classification* (where SOC stands for the 2010 Standard Occupational Classification system), which enables us to uniquely identify all the military personnel ${\mathbf {M}}_v$ in the microfile, $\rho _v = 5{,}519$.

We decided to choose the 5 % sample microfile of the 2000 U.S. Census also available from the IPUMS-International Project (Ruggles et al. 2010) as the auxiliary microfile $\tilde{{\mathbf {M}}}$. This microfile contains $\tilde{\rho } = 6{,}309{,}848$ records. Since this microfile also contains attributes *Place of work: state, 1980 onward* and *Place of work: PUMA, 2000 onward*, we decided to replace them with the *Place of work* attribute in the same way as described above.

This auxiliary microfile satisfies all the necessary requirements:

- records in $\tilde{{\mathbf {M}}}$ and in ${\mathbf {M}}$ are drawn from sufficiently similar distributions under assumption that demographics of respondents in both microfiles haven’t changed much over 13 years;
- $\tilde{{\mathbf {M}}}$ contains an auxiliary vital attribute *Occupation, SOC classification*, identical to the vital attribute in ${\mathbf {M}}$ in terms of military occupations. Vital records in ${\mathbf {M}}$ and auxiliary vital ones in $\tilde{{\mathbf {M}}}$ are drawn from sufficiently similar distributions under assumption that demographics of military personnel haven’t changed much over 13 years. There are $\tilde{\rho } = 19{,}149$ auxiliary vital records in $\tilde{{\mathbf {M}}}_v$;
- ${\mathbf {M}}$ and $\tilde{{\mathbf {M}}}$ contain almost identical attributes, with the exception of several technical ones. In our example, we performed the following harmonization:
  - we replaced the *Occupation, SOC classification* attribute in both microfiles with a new one *Military Personnel*, which has only two values, 0 and 1. The value 1 was assigned only to those records that had one of the values of attribute *Occupation, SOC classification* presented in Table 2;
  - we removed all attributes from both microfiles except for *Military Personnel*, *Place of work*, and $t^H = 13$ basic harmonized attributes, which we consider to be useful for building a fuzzy model of a group.

Information about each basic harmonized attribute $w^H_{b_i}$, $i = 1,2,\ldots , 13$, is given in Table 3, where *C* stands for a categorical attribute, *O* stands for an ordinal one.

**Table 2:** Values of the *Occupation, SOC classification* attribute that correspond to the value 1 of the harmonized attribute *Military Personnel*

Attribute value	Interpretation
551,010	Military Officer Special and Tactical Operations Leaders
552,010	First-Line Enlisted Military Supervisors
553,010	Military Enlisted Tactical Operations and
	Air/Weapons Specialists and Crew Members
559,830	Military, Rank Not Specified

**Table 3:** Basic harmonized attributes used in the practical example

Index	Name	Type	Values
$b_1$	Age	*O*	000—*Less than 1 year old*, $1\dots 130$—1 to 130 years, 135—135
$b_2$	Educational attainment [general version]	*C*	00—*N/A or no schooling*, 01—*Nursery school to grade 4*, 02—*Grade 5, 6, 7, or 8*, 03—*Grade 9*, 04—*Grade 10*, 05—*Grade 11*, 06—*Grade 12*, 07—*1 year of college*, 08—*2 years of college*, 09—*3 years of college*, 10—*4 years of college*, 11—*5+ years of college*
$b_3$	Sex	*C*	1—*Male*, 2—*Female*
$b_4$	Race [general version]	*C*	1—*White*, 2—*Black/Negro*, 3—*American Indian or Alaska Native*, 4—*Chinese*, 5—*Japanese*, 6—*Other Asian or Pacific Islander*, 7—*Other race, nec*, 8—*Two major races*, 9—*Three or more major races*
$b_5$	Usual hours worked per week	*O*	00—*N/A*, $01\ldots 98$—1 to 98 h worked per week, 99—*99 (Topcode)*
$b_6$	Hispanic origin [general version]	*C*	0—*Not Hispanic*, 1—*Mexican*, 2—*Puerto Rican*, 3 —*Cuban*, 4—*Other*, 9—*Not Reported*
$b_7$	Marital status	*C*	1—*Married, spouse present*, 2—*Married, spouse absent*, 3—*Separated*, 4—*Divorced*, 5—*Widowed*, 6—*Never married/single*
$b_8$	Means of transportation to work	*C*	00—*N/A*, 10—*Auto, truck, or van*, 11—*Auto*, 12—*Driver*, 13—*Passenger*, 14—*Truck*, 15—*Van*, 20—*Motorcycle*, 30—*Bus or streetcar*, 31—*Bus or trolley bus*, 32—*Streetcar or trolley car*, 33—*Subway or elevated*, 34—*Railroad*, 35—*Taxicab*, 36—*Ferryboat*, 40—*Bicycle*, 50—*Walked only*, 60—*Other*, 70—*Worked at home*
$b_9$	Time of departure for work	*O*	0000—*N/A*, other values report the time usually leaving for work last week (12:01 a.m. is coded as 0001, and 11:59 p.m. is coded as 2359)
$b_{10}$	Travel time to work	*O*	000—*N/A*, other values are amounts of time, in minutes, it took to get to work last week
$b_{11}$	Weeks worked last year, intervalled	*C*	0—N/A, 1—*1–13 weeks*, 2—*14–26 weeks*, 3—*27–39 weeks*, 4—*40–47 weeks*, 5—*48–49 weeks*, 6—*50–52 weeks*
$b_{12}$	Total personal income	*O*	A 7-digit numeric code reporting each respondent’s total pre-tax personal income or losses from all sources for the previous year
$b_{13}$	Speaks English	*C*	0—*N/A (Blank)*, 1 —*Does not speak English*, 2—*Yes, speaks English...*, 3—*Yes, speaks only English*, 4—*Yes, speaks very well*, 5—*Yes, speaks well*, 6 —*Yes, but not well*, 7—*Unknown*, 8—*Illegible*

### Input variables identification

In this section, we will discuss linguistic variables $L_j$ corresponding to basic harmonized attributes $w^H_{b_j}$, $j = 1,2,\ldots , 13$. Each $L_j$ bares the name of the corresponding attribute $w_{b_j}$. Ranges $\left[ l\left( L_j\right) , u\left( L_j\right) \right]$ of acceptable values of base variables for each $L_j$, $j = 1,2,\ldots , 13$, are given in Table 4.

**Table 4:** Ranges of acceptable for each linguistic variable in the practical example

Name of $L_j$	$l\left( L_j\right)$	$u\left( L_j\right)$
Age	18	45
Educational attainment [general version]	1	11
Sex	1	2
Race [general version]	1	2
Usual hours worked per week	0	100
Hispanic origin [general version]	0	9
Marital status	1	6
Means of transportation to work	0	70
Time of departure for work	1	2359
Travel time to work	1	119
Weeks worked last year, intervalled	1	6
Total personal income	0	200,000
Speaks English	2	5

After having removed all the records, whose basic harmonized attribute values don’t belong to the specified ranges, we obtained the microfiles ${\mathbf {M}}^H$ with $\rho = 565,243$, $\rho _v = 3,992$, and $\tilde{{\mathbf {M}}}^H$ with $\tilde{\rho } = 3,205,478$, $\tilde{\rho }_v = 14,263$.

Let us introduce several generic membership functions of one argument *x* and several parameters:

$$\begin{aligned} GAUSSMF\left( x, a, b\right)&= {} e^{- \frac{\left( x - b\right) ^2}{2a^2}},\\ TRAPMF\left( x, a, b, c, d\right)&= {} \left\{ \begin{array}{cc} 0, &{} x \le a\\ \frac{x - a}{b - a}, \quad &{} a \le x \le b\\ 1, \quad &{} b \le x \le c\\ \frac{d - x}{d - c}, \quad &{} c \le x \le d\\ 0, &{} x \ge d \end{array} \right. ,\\ PIMF\left( x, a, b, c, d\right)&= {} \left\{ \begin{array}{cc} 0, &{} x \le a\\ 2\left( \frac{x - a}{b - a}\right) ^2, \quad &{} a \le x \le \frac{a + b}{2}\\ 1 - 2\left( \frac{x - b}{b - a}\right) ^2, &{} \frac{a + b}{2} \le x \le b\\ 1, &{} b \le x \le c\\ 1 - 2\left( \frac{x - c}{d - c}\right) ^2, \quad &{} c \le x \le \frac{c + d}{2}\\ 2\left( \frac{x - d}{d - c}\right) ^2, &{} \frac{c + d}{2} \le x \le d\\ 0, &{} x \ge d \end{array} \right. . \end{aligned}$$

Then, the fuzzy values of all linguistic variables are as follows:

1. Variable $L_1$ has 5 fuzzy values:
  *Young*, with the membership function
    $$\begin{aligned} \mu _{A_{1,1}}\left( x\right) = PIMF\left( x, 7.05, 15.40, 22.50, 27.18\right) ; \end{aligned}$$
  *Middle Aged 1*, with $\mu _{A_{1,2}}\left( x\right) = GAUSSMF\left( x, 2.0, 27.5\right) ;$
  *Middle Aged 2*, with $\mu _{A_{1,3}}\left( x\right) = GAUSSMF\left( x, 2.0, 32.5\right) ;$
  *Middle Aged 3*, with $\mu _{A_{1,4}}\left( x\right) = GAUSSMF\left( x, 2.0, 37.5\right) ;$
  *Old*, with $$\mu _{A_{1,5}}\left( x\right) = PIMF\left( x, 37.85, 42.50, 47.51, 54.84\right)$$.
2. Variable $L_2$ has 2 fuzzy values:
  *Low*, with $$\mu _{A_{2,1}}\left( x\right) = TRAPMF\left( x, 1, 1, 8, 10\right) ;$$
  *High*, with $$\mu _{A_{2,2}}\left( x\right) = TRAPMF\left( x, 8, 10, 11, 11\right)$$.
3. Variable $L_3$ has 2 fuzzy values:
  *Male*, with $$\mu _{A_{3,1}}\left( x\right) = \left\{ \begin{array}{ll} 1, &{}\quad x = 1\\ 0, &{}\quad \text {otherwise} \end{array} \right.$$
  *Female*, with $$\mu _{A_{3,2}}\left( x\right) = \left\{ \begin{array}{ll} 1, &{}\quad x = 2\\ 0, &{}\quad \text {otherwise} \end{array} \right.$$
4. Variable $L_4$ has 2 fuzzy values:
  *White*, with $$\mu _{A_{4,1}}\left( x\right) = \left\{ \begin{array}{ll} 1, &{}\quad x = 1\\ 0, &{}\quad \text {otherwise} \end{array} \right.$$
  *Black*, with $$\mu _{A_{4,2}}\left( x\right) = \left\{ \begin{array}{ll} 1, &{}\quad x = 2\\ 0, &{}\quad \text {otherwise} \end{array} \right.$$
5. Variable $L_5$ has 3 fuzzy values:
  *Low*, with $$\mu _{A_{5,1}}\left( x\right) = PIMF\left( x, 0.0, 0.0, 29.9, 40.3\right) ;$$
  *Medium*, with $$\mu _{A_{5,2}}\left( x\right) = GAUSSMF\left( x, 2.5, 40.0\right) ;$$
  *High*, with $$\mu _{A_{5,3}}\left( x\right) = PIMF\left( x, 40.2, 50.1, 100.0, 100.0\right)$$.
6. Variable $L_6$ has 2 fuzzy values:
  *No*, with $$\mu _{A_{6,1}}\left( x\right) = \left\{ \begin{array}{ll} 1, &{}\quad x = 0\\ 0, &{}\quad \text {otherwise} \end{array} \right.$$
  *Yes*, with $$\mu _{A_{6,2}}\left( x\right) = \left\{ \begin{array}{ll} 1, &{}\quad 1 \le x \le 9\\ 0, &{}\quad \text {otherwise} \end{array} \right.$$
7. Variable $L_7$ has 2 fuzzy values:
  *Married*, with $$\mu _{A_{7,1}}\left( x\right) = \left\{ \begin{array}{ll} 1, &{}\quad 1 \le x \le 2\\ 0, &{}\quad \text {otherwise} \end{array} \right.$$
  *Not married*, with $$\mu _{A_{7,2}}\left( x\right) = \left\{ \begin{array}{ll} 1, &{}\quad 3 \le x \le 6\\ 0, &{}\quad \text {otherwise} \end{array} \right.$$
8. Variable $L_8$ has 3 fuzzy values:
  *Car*, with $$\mu _{A_{8,1}}\left( x\right) = \left\{ \begin{array}{ll} 1, &{}\quad 0 \le x \le 20\\ 0, &{}\quad \text {otherwise} \end{array} \right.$$
  *Public*, with $$\mu _{A_{8,2}}\left( x\right) = \left\{ \begin{array}{ll} 1, &{}\quad 30 \le x \le 36\\ 0, &{}\quad \text {otherwise} \end{array} \right.$$
  *Walked*, with $$\mu _{A_{8,3}}\left( x\right) = \left\{ \begin{array}{ll} 1, &{}\quad 40 \le x \le 50\\ 0, &{}\quad \text {otherwise} \end{array} \right.$$
9. Variable $L_9$ has 3 fuzzy values:
  *Night*, with $$\mu _{A_{9,1}}\left( x\right) = PIMF\left( x, 1, 1, 530, 630\right) ;$$
  *Morning*, with $$\mu _{A_{9,2}}\left( x\right) = PIMF\left( x, 530, 630, 800, 900\right) ;$$
  *Day*, with $$\mu _{A_{9,3}}\left( x\right) = PIMF\left( x, 800, 900, 2359, 2359\right)$$.
10. Variable $L_{10}$ has 3 fuzzy values:
  *Little*, with $$\mu _{A_{10,1}}\left( x\right) = PIMF\left( x, 1, 1, 10, 15\right) ;$$
  *Medium*, with $$\mu _{A_{10,2}}\left( x\right) = PIMF\left( x, 10, 15, 30, 45\right) ;$$
  *Much*, with $$\mu _{A_{10,3}}\left( x\right) = PIMF\left( x, 35, 45, 120, 120\right)$$.
11. Variable $L_{11}$ has 2 fuzzy values:
  *Abnormal*, with $$\mu _{A_{11,1}}\left( x\right) = TRAMPF\left( x, 1, 1, 5, 6\right) ;$$
  *Normal*, with $$\mu _{A_{11,2}}\left( x\right) = TRAMPF\left( x, 5, 6, 6, 6\right)$$.
12. Variable $L_{12}$ has 3 fuzzy values:
  *Low*, with $$\mu _{A_{12,1}}\left( x\right) = PIMF\left( x, 0, 0, 9000, 12000\right) ;$$
  *Medium*, with $$\mu _{A_{12,2}}\left( x\right) = PIMF\left( x, 9000, 12000, 70000, 90000\right) ;$$
  *High*, with $$\mu _{A_{12,3}}\left( x\right) = PIMF\left( x, 70000, 90000, 200000, 200000\right)$$.

We decided not to define values for variable $L_{13}$. Its range of acceptable values was used to remove unacceptable records from the microfiles, but the attribute itself was not involved in the fuzzy rules evolved using the evolutionary algorithm.

### Generating fuzzy rules by the evolutionary algorithm

In order to evolve fuzzy rules to obtain the auxiliary quantity signal for the practical example, we applied the evolutionary algorithm with the following parameters:

- the population size $\mu$ was fixed at 100;
- on each iteration, we replaced $\lambda = 40$ worst fit individuals with the newly obtained by applying recombination and mutation operators;
- we applied recombination operator with the probability $p_c = 1.00$, and mutation operator with probability $p_m = 0.05$;
- we performed 10 separate runs of the evolutionary algorithm, each of which lasted for $N = 100$ generations.

Of all the fuzzy rules obtained in all generations, we selected the fuzzy rules, whose RCF was greater than $\gamma =0.750$ and support was greater than $\kappa =0.001$. After that, we removed those rules that are more specific versions of the more general ones in the set, as described previously. In Table 5, we presented all of the resultant rules. For each fuzzy rule from the rule base, we specified its discriminative factor, relative confidence factor, and support. We present all the numerical values with 3 significant numbers, although the calculations were carried out with a much higher precision.

**Table 5:** Fuzzy rules used in the example

*R*	*DF*	*RCF*	Support
$\left( 1, 0, 0, 0, 0, 0, 2, 3, 1, 1, 0, 2\right)$	0.032	0.755	0.032
$\left( 1, 0, 0, 0, 3, 0, 0, 3, 1, 0, 0, 0\right)$	0.031	0.787	0.031
$\left( 1, 0, 0, 0, 3, 1, 0, 3, 0, 0, 2, 1\right)$	0.012	0.801	0.012
$\left( 1, 0, 0, 1, 0, 1, 0, 3, 1, 1, 1, 2\right)$	0.010	0.781	0.010
$\left( 1, 0, 0, 1, 3, 0, 0, 3, 0, 0, 2, 1\right)$	0.012	0.851	0.012
$\left( 1, 0, 1, 0, 0, 0, 0, 3, 1, 1, 0, 2\right)$	0.034	0.840	0.034
$\left( 1, 0, 1, 0, 0, 0, 2, 3, 1, 1, 2, 0\right)$	0.025	0.765	0.025
$\left( 1, 0, 1, 0, 3, 0, 2, 3, 2, 0, 0, 1\right)$	0.018	0.931	0.018
$\left( 1, 0, 1, 0, 3, 1, 0, 3, 2, 0, 0, 1\right)$	0.017	0.915	0.018
$\left( 1, 0, 1, 1, 0, 0, 0, 3, 1, 1, 2, 0\right)$	0.025	0.754	0.026
$\left( 1, 0, 1, 1, 0, 0, 2, 3, 1, 0, 0, 2\right)$	0.032	0.751	0.032
$\left( 1, 1, 0, 0, 3, 0, 2, 3, 2, 1, 0, 1\right)$	0.018	0.951	0.018
$\left( 1, 1, 0, 0, 3, 1, 0, 3, 2, 0, 0, 1\right)$	0.019	0.767	0.019
$\left( 1, 1, 1, 0, 3, 0, 0, 3, 2, 0, 2, 1\right)$	0.009	1.876	0.009
$\left( 1, 1, 1, 0, 3, 0, 0, 3, 2, 1, 1, 1\right)$	0.008	0.761	0.009
$\left( 1, 1, 1, 0, 3, 0, 2, 3, 0, 1, 2, 1\right)$	0.010	1.325	0.010
$\left( 1, 1, 1, 0, 3, 0, 2, 3, 2, 1, 2, 0\right)$	0.026	0.767	0.026
$\left( 1, 2, 1, 0, 0, 0, 0, 3, 1, 0, 0, 2\right)$	0.002	0.914	0.002

As we can see, all of these rules share one common characteristic, i.e., their value of variable $L_8$ is *Walked*, which means that all the respondents considered by the fuzzy rules as military personnel walked to their work rather than used a car or other means of transportation. Judging from the values of other variables, we can make general conclusions that these respondents typically are young males with medium yearly income.

### Disclosing outliers in the group distribution using evolved fuzzy rules

To demonstrate how the evolved fuzzy rules can be used to violate outliers in the quantity signal, we will first apply them to the auxiliary microfile, and then proceed to disclosing outliers in quantity signals obtained for the main microfile.

Since it would be impractical to try to analyze the auxiliary quantity signal constructed for all the PUMAs as a whole (there were 1238 different PUMAs circa 2000 in the U.S.), we will present appropriate results state by state.

**Fig. 1:**
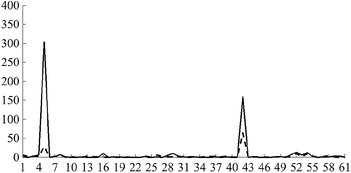
Quantity signal (*solid line*) and auxiliary quantity signal (*dashed line*) obtained for the state of New York by applying the fuzzy rules from the example to the 2000 U.S. census microfile

Let us consider for illustration purposes the state of New York. In Fig. 1, we presented both the quantity signal (solid line) and the auxiliary quantity signal (dashed line). Values $i = 1,2,\ldots , 61$ over the *x* axis stand for the $i\hbox {th}$ PUMA of the state of New York. The list of PUMAs can be found on the IPUMS-International website (PUMAs and Super-PUMAs 2000) . The values over the *y* axis stand for:

- in case of the quantity signal, the number of military personnel working in a corresponding PUMA;
- in case of the auxiliary quantity signal, the sum of all membership grades assigned to the respondents in a corresponding PUMA by the evolved fuzzy rules.

Applying MTTT with $\alpha = 0.01$ to the quantity signal, we can obtain the following index set:

$$\begin{aligned} OUT\left( {\mathbf {q}}_{NY\ 2000}\right) = \left\{ 3,4,5,7,8,16,24,28,29,30,42,44,49,51,\ldots ,55,59,60\right\} . \end{aligned}$$

Analysis of the Report of the Deputy Under Secretary of Defense (2000) permits us to conclude that most of the indexes obtained by MTTT do not correspond to sites of military bases. Further on, we will assume that $OUT_e\left( {\mathbf {q}}_{NY\ 2000}\right) = \left\{ 5, 42\right\}$, because:

- the outlier in PUMA 5 corresponds to *Fort Drum* (Deputy Under Secretary of Defense 2000, p. ARMY-9);
- the outlier in PUMA 42 corresponds to *West Point Military Reservation* (Deputy Under Secretary of Defense 2000, p. ARMY-10).

Applying MTTT with $\alpha = 0.01$ to the auxiliary quantity signal, we can obtain the following index set:

$$\begin{aligned} OUT\left( {\mathbf {q}}^{aux}_{NY\ 2000}\right) = \left\{ 1,5,8,26,36,42,52,54,58\right\} . \end{aligned}$$

Taking into account previous discussion, we can assume that $OUT_e\left( {\mathbf {q}}^{aux}_{NY\ 2000}\right) = \left\{ 5, 42\right\}$. Equality $OUT_e\left( {\mathbf {q}}_{NY\ 2000}\right) = OUT_e\left( {\mathbf {q}}^{aux}_{NY\ 2000}\right)$ indicates that the sites of military facilities can be easily disclosed even if the vital attributes are removed from the microfile.

In a similar fashion, we can analyze all the other states and determine undisclosed and false outliers. The overall figures are given in Table 6. We included in the table only those states, where the number of working military personnel exceeds 0.5 % of all the military personnel in original harmonized auxiliary microfile $\tilde{{\mathbf {M}}}^H$, i.e., the value $95.245 = 00.005 \cdot 19,049$.

**Table 6:** Results of applying the evolved fuzzy rules to the 2000 census microfile

State	Number of outliers in the quantity signal	Number of undisclosed outliers	Number of outliers in the auxiliary quantity Signal	Number of false outliers
Alabama	4	3	1	0
Alaska	2	0	2	0
Arizona	4	1	3	0
California	4	0	4	0
Colorado	2	0	2	0
Connecticut	1	0	1	0
Florida	7	4	4	1
Georgia	5	1	5	1
Hawaii	1	0	1	0
Illinois	2	1	1	0
Kansas	3	2	1	0
Kentucky	2	0	2	0
Louisiana	4	2	2	0
Maryland	2	1	1	0
Mississippi	2	1	1	0
Missouri	2	1	1	0
New Jersey	3	1	2	0
New York	2	0	2	0
North Carolina	4	2	2	0
Ohio	4	3	1	0
Oklahoma	3	2	1	0
Pennsylvania	4	2	4	2
Rhode Island	1	0	1	0
South Carolina	6	1	5	0
Tennessee	3	3	0	0
Texas	7	2	5	0
Virginia	9	3	6	0
Washington	5	2	3	0
Total	98	38	64	4

The confusion matrix (18) for this example is

$$\begin{aligned} {\mathbf {Z}} = \left( \begin{array}{cc} 60 &{} 38\\ 4 &{} 785 \end{array}\right) . \end{aligned}$$

The tests (19), (20), and (22) based on the values of ${\mathbf {Z}}$ are as follows: $PA = 0.953$, $J = 0.891$, $MB = 98.047$. These figures indicate the high effectiveness of the evolved fuzzy rules in disclosing sensitive data features.

**Fig. 2:**
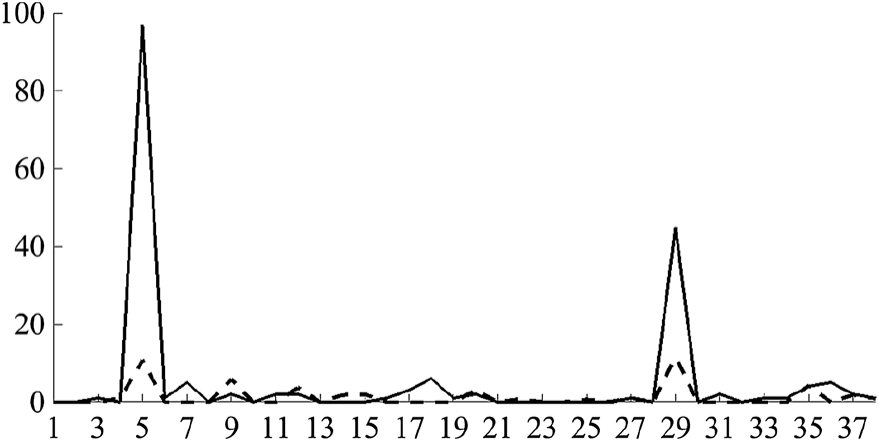
Quantity signal (*solid line*) and auxiliary quantity signal (*dashed line*) obtained for the state of New York by applying the fuzzy rules from the example to the 2013 U.S. ACS microfile

Let us now discuss the results of the application of the evolved fuzzy rules to the original microfile ${\mathbf {M}}^H$. In Fig. 2, we presented both quantity signal (solid line) and auxiliary quantity signal (dashed line) for the state of New York. Values $i = 1,2,\ldots , 38$ over the *x* axis stand for the $i\hbox {th}$ PUMA of the state of New York. The list of PUMAs circa 2013 can be found on the IPUMS-International website (PUMAs 2010).

Applying MTTT with $\alpha = 0.01$ to the quantity signal, we can obtain the following index set:

$$\begin{aligned} OUT\left( {\mathbf {q}}_{NY\ 2013}\right) = \left\{ 5,7,9,11,12,17,18,20,29,31,35,36,37\right\} . \end{aligned}$$

As pointed out before, analysis of (Deputy Under Secretary of Defense 2000) permits us to conclude that most of the indexes obtained by MTTT do not correspond to sites of military bases. Further on, we will assume that $OUT_e\left( {\mathbf {q}}_{NY\ 2013}\right) = \left\{ 5, 29\right\}$, because:

- the outlier in PUMA 5 corresponds to *Fort Drum* (Deputy Under Secretary of Defense 2000, p. ARMY-9);
- the outlier in PUMA 42 corresponds to *West Point Military Reservation* (Deputy Under Secretary of Defense 2000, p. ARMY-10).

Applying MTTT with $\alpha = 0.01$ to the auxiliary quantity signal, we can obtain the following index set:

$$\begin{aligned} OUT\left( {\mathbf {q}}^{aux}_{NY\ 2013}\right) = \left\{ 4,5,9,12,14,15,20,22,25,27,29,35,37,38\right\} . \end{aligned}$$

Taking into account previous discussion, we can assume that $OUT_e\left( {\mathbf {q}}^{aux}_{NY\ 2013}\right) = \left\{ 5, 29\right\}$. I.e., both outliers are clearly visible in the auxiliary quantity signal as well.

Analogous results for other states are given in Table 7. We once again included in the table only those states, where the number of working military personnel exceeds 0.5 % of all the military personnel in original harmonized microfile ${\mathbf {M}}^H$, i.e., the value $27.595 = 0.005 \cdot 5,519$.

**Table 7:** Results of applying the evolved fuzzy rules to the 2013 ACS microfile

State	Number of outliers in the quantity signal	Number of undisclosed outliers	Number of outliers in the auxiliary quantity signal	Number of false outliers
Alabama	2	2	1	1
Alaska	2	0	2	0
Arizona	4	1	4	1
California	3	1	2	0
Colorado	2	0	2	0
Connecticut	1	0	2	1
Florida	7	5	3	1
Georgia	7	3	4	0
Hawaii	1	0	1	0
Illinois	2	1	2	1
Kansas	2	2	0	0
Kentucky	2	1	1	0
Louisiana	4	4	0	0
Maryland	3	2	1	0
Mississippi	1	0	1	0
Missouri	2	2	0	0
Nevada	1	0	1	0
New Jersey	2	2	0	0
New Mexico	2	2	0	0
New York	2	0	2	0
North Carolina	3	1	2	0
Ohio	2	1	3	2
Oklahoma	3	2	1	0
South Carolina	4	1	3	0
Texas	6	1	5	0
Virginia	7	4	4	1
Washington	4	1	3	0
Total	81	39	50	8

The confusion matrix (18) for this example is

$$\begin{aligned} {\mathbf {Z}} = \left( \begin{array}{cc} 42 &{} 39\\ 8 &{} 564 \end{array}\right) . \end{aligned}$$

The tests (19), (20), and (22) based on the values of ${\mathbf {Z}}$ are as follows: $PA = 0.930$, $J = 0.775$, $MB = 55.067$. The values of all the tests are lower than their counterparts calculated for the 2000 census data. The matter is that the fuzzy rules were evolved using 2000 census data. Nevertheless, presented values indicate high effectiveness of the evolved fuzzy rules and their good generalization abilities.

### Results of protecting group distributions using memetic algorithm

**Fig. 3:**
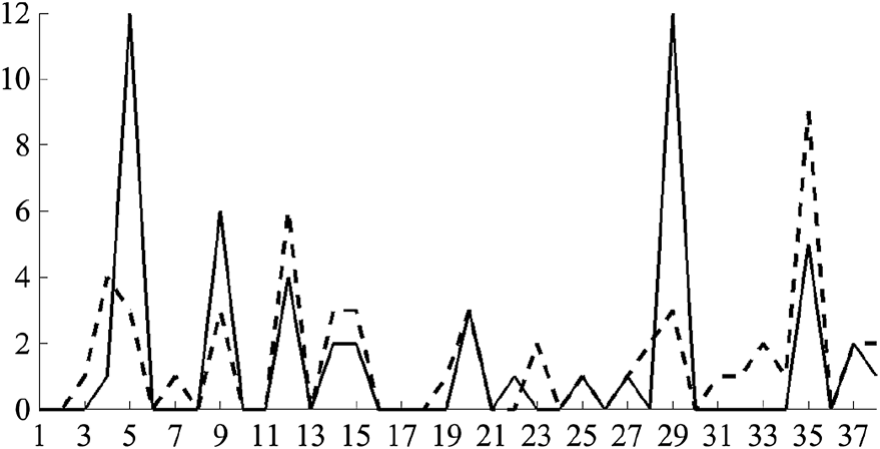
Initial (*solid line*) and modified (*dashed line*) auxiliary quantity signals for the state of New York (2013 U.S. ACS microfile)

To illustrate the application of the MA for protecting outliers in the auxiliary quantity signal for the task discussed above, we will limit ourselves to the state of New York. There is a total of 91,398 respondents that work in this state. The auxiliary quantity signal corresponding to this state is shown in Fig. 2 (dashed line), and the corresponding crisp auxiliary quantity signal is shown in Fig. 3 (solid line).

As we’ve already discussed earlier, to mask the outliers in the signal, we need to reduce the values of the $5\hbox {th}$ and the $29\hbox {th}$ signal elements. We can achieve this task by imposing such fuzzy restrictions that lead the evolutionary process in the direction of obtaining signals, whose $5\hbox {th}$ and $29\hbox {th}$ signal values will not be greater than 2.

This leads to the following fitness function:

28 $$\begin{aligned} f\left( U\right)&= {} \frac{299 - \sum _{i = 1}^Q \sum _{k = 1}^{13}\text {sign}\left| {\mathbf {M}}_{u_{i1}}\left( u_{i2}, W_k\right) - {\mathbf {M}}_{u_{i3}}\left( u_{i4}, W_k\right) \right| }{299} \nonumber \\&\cdot \, ZMF\left( q_5^{*}\left( U\right) , 2, 12\right) \cdot ZMF\left( q_{29}^{*}\left( U\right) , 2, 12\right) \nonumber \\&\cdot \, \frac{1}{1 + e^{\frac{1}{2}\left( Q_U - 25\right) }}, \end{aligned}$$

where $C_{\max } = 299$; $W_k$, $k = 1,2,\ldots , 13$, is the $k\hbox {th}$ basic attribute; ${\mathbf {M}}_j\left( i, W_k\right)$ returns the value of the attribute $W_k$ of the $i\hbox {th}$ record in ${\mathbf {M}}_j$; $ZMF\left( x, a, b\right)$ is a function defined as

$$\begin{aligned} ZMF\left( x, a, b\right) = \left\{ \begin{array}{ll} 1, &{}\quad x \le a\\ 1 - 2\left( \frac{x - a}{b - a}\right) ^2, &{}\quad a \le x \le \frac{a + b}{2}\\ 2\left( \frac{x - b}{b - a}\right) ^2, &{}\quad \frac{a + b}{2} \le x \le b\\ 0, &{}\quad x \ge b \end{array} \right. . \end{aligned}$$

Each row in (28) corresponds to a single part of the fitness function (26).

To simplify the matters, we considered all the basic attributes to be categorical ones with following parameters of (25): $\gamma _k = 1 \; \forall k = 1,2,\ldots , 13$, $\chi _1 = 1$, $\chi _2 = 0$. The metric (25) defined this way shows the number of attribute values that need to be physically altered during one swap of the records between the submicrofiles.

We decided to apply tournament selection (Brindle 1981) as an efficient and easy to implement selection operator, with the tournament size 5. Other algorithm parameters were chosen as follows: $\mu = 100$, $\lambda = 40$, $p_c = 1$, $p_{m_1} = p_{m_2} = p_{m_3} = p_{m_4} = 0.001$, $p_{mem} = 0.75$. We terminated the algorithm after having obtained 1000 consequent populations.

The population was initialized by randomly generating matrices with different numbers of rows. Elements of the first column were generated with probabilities proportional to the values of the corresponding elements of ${\mathbf {q}}$. Elements of the third column were generated with probabilities proportional to the total numbers of records in corresponding submicrofiles.

During the MA run, we applied linear fitness scaling in the form presented in Goldberg (1989, p. 79) to prevent premature convergence. We also multiplied the mutation probabilities by the factor of 10 whenever the standard deviation of the population fitness values dropped below 0.03.

We performed 10 runs of the MA. Among 1000 individuals obtained in the last generations of each run, 983 correspond to valid solutions of the TPGA in terms of masking outliers in the auxiliary quantity signal. In Fig. 3 (dashed line), we presented the solution with the lowest cumulative influential metric (25), namely, 53. This solution is valid because applying MTTT to it yields $OUT\left( {\mathbf {q}}^{aux*}_{NY\ 2013}\right) = \left\{ 12, 35\right\}$. Since $OUT_e\left( {\mathbf {q}}^{aux}_{NY\ 2013}\right) \cap OUT\left( {\mathbf {q}}^{aux*}_{NY\ 2013}\right) = \emptyset$, we can conclude that the memetic algorithm managed to successfully modify the auxiliary quantity signal by creating new outliers in the $35\hbox {th}$ and $12\hbox {th}$ signal elements and eliminating the real ones.

The mean cumulative metric (25) over all solutions that can be in a similar fashion viewed as valid is 62.518, i.e., we need to alter only $\frac{62.518}{13\cdot 1,380,924} \approx 0.0003$ % of microfile attribute values in order to provide group anonymity.

## Conclusions

In this work, we demonstrated that even if vital attributes are removed from the microfile, it does not necessarily follow that group anonymity is fully provided. Using an appropriately tailored evolutionary algorithm, it is possible to build up the fuzzy model of a group in the form of fuzzy rules that can violate group anonymity. We also discussed how memetic algorithms can be used to really provide group anonymity in a microfile at the cost of introducing only a small amount of distortion into the micro data.

Much work remains to be done. Several directions for future research include: enhancing the classification accuracy of the fuzzy rules and enhancing the memetic algorithm efficiency by choosing appropriate operators.
